# Spontaneous Pneumothorax in an Allogeneic Cell Transplant Recipient with Invasive Pulmonary Aspergillosis and Antecedent RSV Pneumonitis.

**DOI:** 10.4084/MJHID.2011.014

**Published:** 2011-04-01

**Authors:** Liang-Piu Koh, Michelle Li-Mei Poon, John Kit-Chung Tam, Lynette Teo, Li-Yang Hsu

**Affiliations:** 1 Departments of Hematology-Oncology; 2 Cardiothoracic and Vascular Surgery; 3 Diagnostic Imaging and; 4 Medicine, National University Health System, Singapore

## Abstract

We report a case of invasive pulmonary aspergillosis (IPA) following respiratory syncytial virus infection in an allogeneic hematopoietic stem cell transplant (HSCT) recipient with chronic graft-versus-host disease. Delayed diagnosis of IPA resulted in the development of a pneumothorax, a rare consequence of fungal pneumonia. Respiratory virus infections are often harbingers of other infective organisms in HSCT recipients. More aggressive diagnostic investigations such as computed tomography scans of the thorax and bronchoscopy with bronchoalveolar lavage should be considered early in any HSCT patient presenting with respiratory virus pneumonia, particularly if atypical features are present or recovery is delayed.

## Introduction:

Pulmonary complications develop in 40% to 60% of hematopoietic cell transplant (HSCT) recipients and are recognized as major causes of morbidity and mortality.^1^ The complications are more frequent in allogeneic recipients, especially those with graft versus host disease (GVHD). Pulmonary infiltrates in such patients pose major challenges for clinicians because of the wide differential diagnoses of both infectious and noninfectious conditions.

Community-acquired respiratory viruses (CRVs) such as respiratory syncytial virus (RSV), parainfluenza, and influenza viruses are important causes of acute infectious pulmonary complications, occurring in 15% to 30% of HSCT recipients each year. One-third to one-half of these is due to RSV. The increased mortality seen in RSV infections in this patient population is attributed to both its propensity for causing life-threatening infections in HSCT recipients as well as to a long-term decline in respiratory function in survivors of acute infection.^2^ In addition, infection by RSV has also been implicated in predisposing patients to infections by fungal pathogens such as *Aspergillus* spp.^2^ We present a case of an allogeneic HSCT recipient with GVHD who developed cavitary invasive pulmonary aspergillosis (IPA) following RSV-associated pulmonary injury and acute respiratory failure late after engraftment.

### Case Report

A 40-year-old male of Chinese ethnicity received allogeneic filgastrim-mobilised peripheral blood HSCT from his HLA 6/6 matched sister using reduced-intensity conditioning regimen. He was in his first complete remission from underlying B-cell prolymphocytic leukemia that had been diagnosed 6 months earlier. The course of transplantation was complicated by steroid-refractory grade III acute GVHD of the liver that began 7 months post-transplantation, requiring intensive immunosuppressive therapy with prednisolone followed by subsequent addition of other immunosuppressive agents including tacrolimus, mycopheolate mofetil and sirolimus for steroid-sparing purposes. He continued to receive dapsone and acyclovir prophylaxis, but was not on any antifungal prophylaxis.

**Figure 1:**
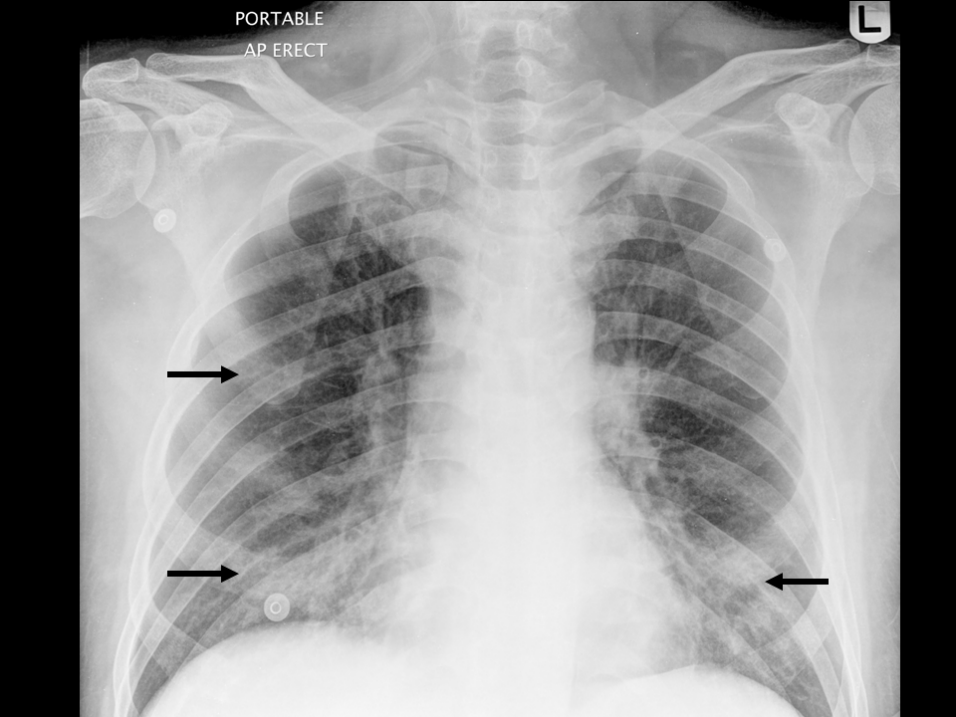
Chest X-ray with black arrows showing patchy air-space changes in both lower zones and the right upper zone.

In December 2007, at 9 months post-transplantation, he presented with acute-onset dry cough and shortness of breath following close contact with a client who had an upper respiratory tract infection (URTI). The initial chest X-ray was unremarkable with no evidence of pneumonia. His symptoms worsened over the ensuing few days, culminating one week later in acute respiratory failure requiring hospitalization and non-invasive mechanical ventilation in the intensive care unit (ICU). The patient was afebrile, with bilateral respiratory rhonchi on chest auscultation. Laboratory studies showed normal blood counts, renal and liver function. A chest x-ray (Figure 1) showed patchy bilateral lower zone and right upper zone air-space changes.

**Figure 2:**
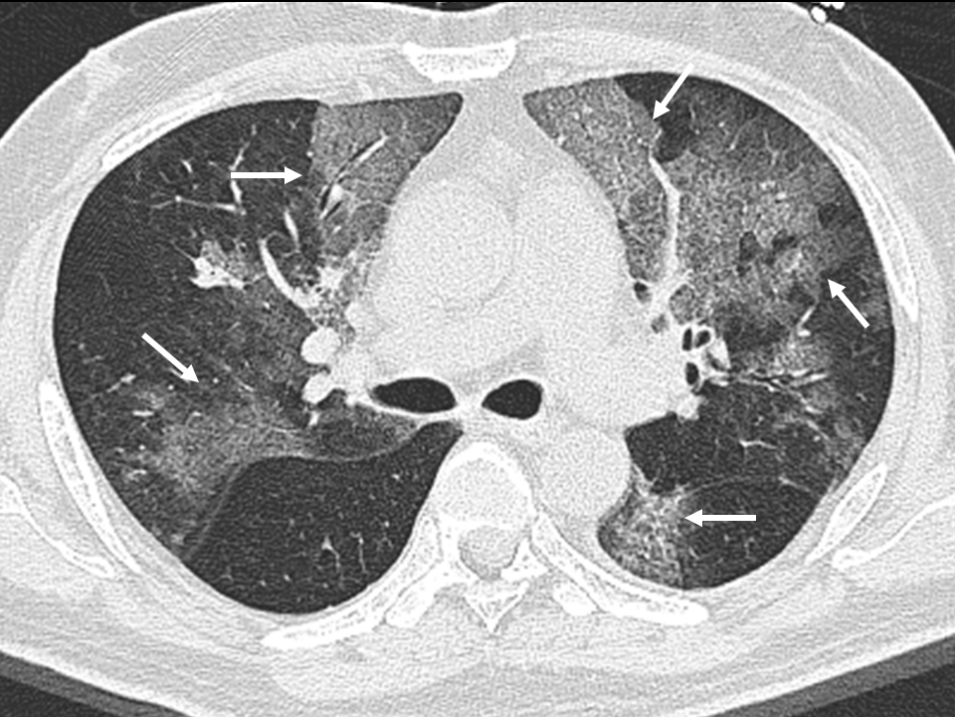
Non-contrast high resolution computed tomography axial image of the thorax with white arrows showing bilateral, circumscribed areas of ground-glass opacification in both lungs with associated reticular changes.

High resolution computed tomography (HRCT) of the thorax (Figure 2) showed bilateral extensive, circumscribed areas of ground-glass opacification throughout both lung fields associated with a fine reticular pattern in keeping with pneumonitis. There were no nodules or pleural-based lesions to suggest a concomitant invasive fungal infection. Empirical antimicrobial therapy with intravenous levofloxacin and trimethoprim/sulfamethoxazole was prescribed for community-acquired bacterial pneumonia and possible pneumocystis jiroveci pneumonitis. Oseltamivir (Tamiflu) was added as the possibility of influenza-associated pneumonia was entertained.

All microscopy and cultures of clinical samples were negative for aerobic bacteria, acid-fast bacilli or fungi. Reverse-transcriptase polymerase chain reactions (PCR) performed on nasopharyngeal specimens were positive for RSV, although viral culture from the same swab was negative. Serial serum galactomannan screening and quantitative real-time PCR for cytomegalovirus DNA from peripheral blood were all negative. Diagnostic bronchoalveolar lavage (BAL) was not performed at this point owing to the concern of worsening hypoxemia.

**Figure 3:**
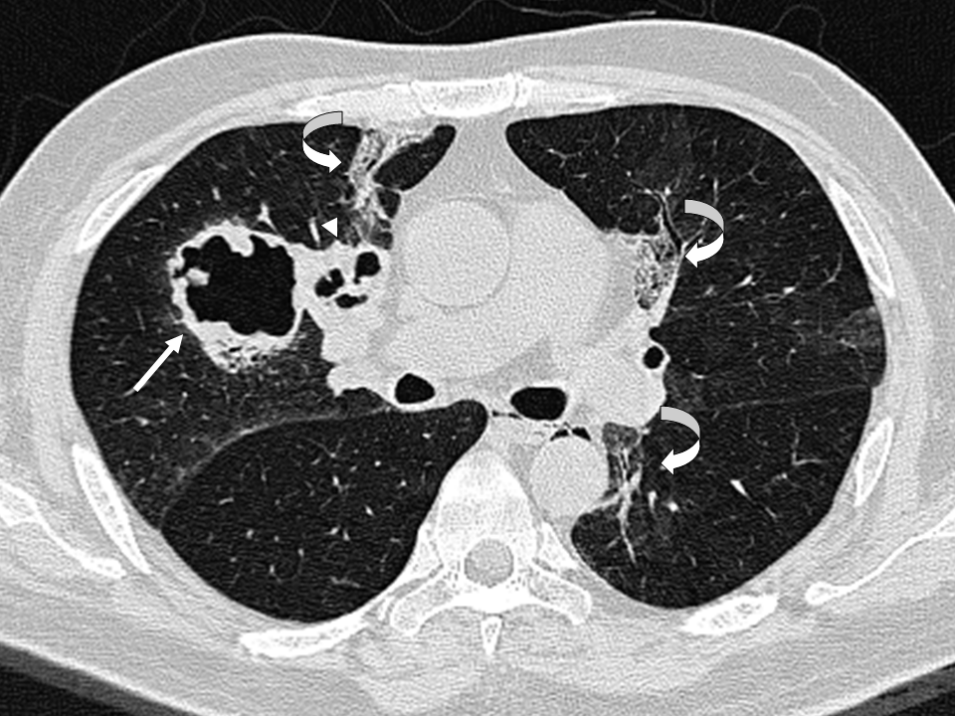
Non-contrast high resolution computed tomography axial image of the thorax showing an irregular thick-walled right upper lobe cavitary lesion (arrow) with adjacent multiseptated cavitary lesion (arrowhead). Faint bilateral circumscribed areas of ground glass opacification are seen in both lungs with associated reticulated chages (curved arrows).

The patient’s overall condition improved gradually over one week, with reduced oxygen requirement and dyspnea. However, a repeat HRCT thorax (Figure 3) 4 weeks later showed new cavitary lesions. Nonetheless, a conservative approach of close clinical monitoring and radiological follow-up was adopted as the serial serum galactomannan tests remained negative and the patient’s overall clinical condition continued to improve. He was discharged one week following the second HRCT.

**Figure 4:**
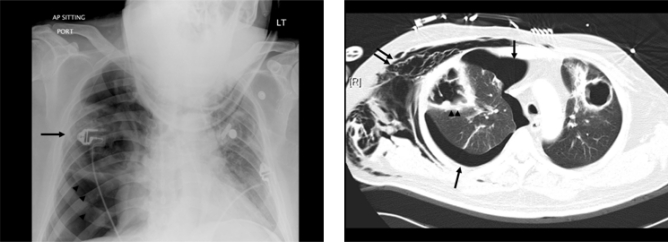
Chest X-ray (A) and contrast-enhanced computed tomography (CT) axial image of the thorax (B) showing right-sided pneumothorax with bilateral thick-walled cavities. The subcutaneous emphysema seen on CT is due to the presence of a right-sided chest drain (not shown).

However, he was re-admitted 10 days later with acute onset right pleuritic chest pain, dyspnea and diaphoresis. Chest x-ray (Figure 4A) and CT thorax (Figure 4B) showed enlarged thick-walled cavitary lesions in both lungs, with a moderate-sized right pneumothorax. Following chest tube insertion, he was transferred to the ICU for invasive mechanical ventilation. Thoracotomy with right upper lobectomy was subsequently performed. The histology was consistent with IPA, while lung tissue cultures grew *Aspergillus fumigatus*. He was prescribed voriconazole, which was continued for 6 months. Serial CT thorax showed gradual regression of the left pulmonary cavitary lesion and complete resolution of the ground grass changes. His respiratory symptoms improved despite receiving continued immunosuppressive therapy for his ongoing extensive chronic GVHD.

## Discussion:

IPA and respiratory syncytial virus (RSV) pneumonia are important causes of morbidity and mortality in allogeneic HCT recipients. The presence of GVHD and immunosuppression are the main predisposing risk factors for the development of both conditions.^1^–^3^

RSV infection in HSCT patients generally presents with the signs and symptoms of a URTI. This may progress to bronchiolitis, pneumonitis, and pneumonia. The frequency of progression of URTI to pneumonia was highest in patients who were <1 month post-transplant or pre-engraftment.^2^ Late onset RSV pneumonia is uncommon, but previous reports, as also illustrated by our case, suggest that chronic GVHD may be a potential risk factor.^2^

The clinical pulmonary syndromes associated with IPA in allogeneic HSCT recipients are generally similar and usually rapidly progressive, with initial symptoms being that of fever, chest pain, cough, and/or hemoptysis. Respiratory failure can develop with progressive growth of mould and inflammation, which can result in necrotic debris and pulmonary infarction.^3^ Spontaneous pneumothorax resulting from IPA is rare.^4^,^5^

The diagnosis of IPA is often made late in the course of disease due to the lack of sensitive and reliable diagnostic techniques. The clinical manifestations are often non-specific. While radiological findings can be suggestive, none are pathognomonic, and cultures of respiratory samples lack sensitivity.^1^,^3^ Recently, attention has been focused on improving diagnostic methods for IPA, including the use of the galactomannan enzyme immunoassay on BAL fluid, which appears to be more sensitive and specific than serum galactomannan assay.^6^ Our patient’s diagnosis of IPA was made only after resection of the affected lung. It remains unknown whether an earlier diagnostic BAL performed during the acute RSV event could have led to an earlier diagnosis of IPA. The enthusiasm in performing BAL in patients with acute respiratory failure, as in this case, has been tempered by life-threatening complications associated with the procedure, such as worsening of hypoxemia and respiratory failure.^7^

Our case lends weight to previously published reports suggesting that respiratory virus infections may play a role in the pathogenesis of bacterial or fungal pneumonia in HSCT recipients.^3^,^8^,^9^ In a retrospective analysis of a cohort of such patients, Nichols and coworkers demonstrated the presence of co-pathogens in 53% of 55 cases of parainfluenza-3 lower respiratory tract infections.^8^ *Aspergillus fumigatus* was the most common co-pathogen, accounting for 13 of the 29 patients with dual infections, and was associated with higher mortality rate. In another report from the same center, Marr and co-workers showed that patients with respiratory virus infections after day 40 post-HSCT also had a 2.1-fold increased risk of subsequent IPA.^9^

Mechanisms that can explain the association between respiratory virus and fungal infections have not been elucidated. Potential contributing factors include GVHD and the immunosuppressive agents used to treat this condition such as steroids and calcineurin inhibitors. It is possible that RSV may itself predispose patients to such infections by damaging the respiratory epithelium and allowing other organisms to colonize and invade. Other potential mechanisms include the immune-modulating effects of the virus itself: RSV can impair multiple antimicrobial effector mechanisms, including inhibition of effector T-cell function as well as immunologic memory development in the respiratory tract, and also diminished microbicidal activity of alveolar macrophages.^2^

In conclusion, our case provides further evidence regarding the direct association between RSV infections and aspergillosis in HCT recipients. As respiratory virus infections are often harbingers of other infective organisms, more aggressive diagnostic investigation such bronchoscopy with BAL should be considered early in any HCT patients presenting with respiratory virus pneumonia. Further studies regarding the interaction of RSV with other post-transplant infections are needed.
